# Detecting limbic predominant neurodegenerative co-pathologies in vivo in Alzheimer’s disease: magnetic resonance imaging markers, cognitive correlates, and prognosis

**DOI:** 10.1186/s13195-025-01885-6

**Published:** 2025-10-30

**Authors:** Nils Richter, Oezguer A. Onur, Gereon R. Fink

**Affiliations:** 1 Cognitive Neuroscience, Institute of Neuroscience and Medicine (INM-3), Research Center Jülich, Jülich, Germany; 2 Department of Neurology, University Hospital Cologne and Faculty of Medicine, University of Cologne, Cologne, Germany

**Keywords:** Limbic predominant age-related TDP-43 encephalopathy (LATE), Argyrophilic grain disease, Dementia, Mild cognitive impairment, Voxel-based morphometry, Tau-protein, Braak stages

## Abstract

**Background:**

In Alzheimer’s disease (AD), limbic non-AD co-pathologies are common and contribute to memory impairment and accelerated clinical progression. To date, in vivo biomarkers of these co-pathologies are lacking. Here, we examined whether specific regional gray matter (GM) atrophy patterns on magnetic resonance imaging (MRI) allow distinguishing between patients with ‘pure’ AD pathology and those with AD pathology and limbic non-AD co-pathologies (AD^+^).

**Methods:**

Atrophy patterns based on ante-mortem MRI scans of histopathologically confirmed ‘pure’ AD (*n =* 36) and AD^+^, i.e., AD pathology with concomitant limbic TDP-43 pathology and argyrophilic grain disease (*n =* 39), were applied to classify an independent cohort of clinically diagnosed patients with mild cognitive impairment (MCI, *n =* 224) and dementia (*n =* 221). Furthermore, we examined the degree to which an MRI marker of cortical degeneration reflecting tau pathology could improve the estimation of clinical progression.

**Results:**

Histopathologically confirmed AD^+^ pathology was associated with more substantial hippocampal atrophy but less cortical degeneration in intermediate Braak stage regions than ‘pure’ AD pathology. Clinically diagnosed AD patients with an AD^+^-classified ratio of cortical-to-hippocampal GM exhibited significantly more memory impairment. At the stage of MCI, AD^+^-classified atrophy was also associated with speeded clinical decline. Furthermore, tau-associated cortical degeneration emerged as the primary predictor of clinical progression across groups and disease stages.

**Conclusions:**

The data suggest that in MCI due to AD, additional non-AD limbic co-pathologies are associated with greater hippocampal but less cortical atrophy and more rapid clinical decline. In contrast, in mild dementia due to AD, hippocampal GM was not associated with prognosis. Instead, cortical degeneration appeared to drive clinical progression.

## Introduction

Alzheimer’s disease (AD) typically first presents with slowly progressive memory decline and is biologically defined by the pathological accumulation of amyloid and tau protein [1]. However, histopathological findings from patients with AD have made it increasingly clear that limbic non-AD co-pathologies frequently occur in AD [2–4].

Clinically, patients with AD and those with AD and limbic co-pathologies, such as limbic predominant age-related TDP-43 encephalopathy (LATE) and argyrophilic grain disease (AGD), are often indistinguishable. However, longitudinal cohort studies with post-mortem histopathological examinations indicate that the progression of cognitive decline and impairment of activities of daily living are much more rapid in patients with mixed pathology than with ‘pure’ AD pathology [5–8], impacting prognosis, clinical management, and everyday life.

Besides, it is unclear to what degree non-AD co-pathologies affect the efficacy of AD-pathology targeting therapies. For example, the presence of non-AD co-pathology may dilute the therapeutic effects of cholinergic or anti-amyloid therapies. On the other hand, the additional detrimental effect of limbic non-AD co-pathologies in AD patients could cause symptoms to emerge earlier, leading to an AD diagnosis when the underlying AD pathological change is still less pronounced than in symptomatic patients with ‘pure’ AD pathology [9]. This, too, could be relevant in the context of anti-amyloid therapies: Evidence suggests that these treatments may be more effective in patients with less severe AD tau pathology [10]. Thus, it cannot be ruled out that, even though AD patients with non-AD co-pathologies generally have a more rapid clinical progression, they could still benefit significantly from an anti-amyloid therapy since their AD pathology might be less severe at the time of diagnosis.

The pathological hallmarks of AD, amyloid and tau pathology, are readily identified in vivo in the routine clinical setting using cerebrospinal fluid (CSF) diagnostics or positron emission tomography (PET). In stark contrast, in vivo molecular biomarkers of limbic non-AD pathologies are lacking [3, 11]. Efforts have been made to operationalize the limbic non-AD (co-) pathologies clinically as limbic amnestic neurodegenerative syndromes (LANS, [12]). Further, imaging markers, such as the inferior to medial temporal lobe ratio of glucose metabolism or gray matter (GM) [2, 13, 14], have been proposed to discriminate between patients with ‘pure’ AD and ‘pure’ non-AD pathologies with reasonable accuracy. However, these markers have shown limited utility in distinguishing between ‘pure’ AD pathology and mixed AD and non-AD limbic pathologies [14].

We aimed to derive a magnetic resonance imaging (MRI) biomarker for detecting non-AD co-pathology in patients with AD from post-mortem histopathological data and to test the prognostic value of such a marker regarding clinical progression in an independent sample. Specifically, the goal was to examine this in patients at the stage of mild cognitive impairment (MCI) or mild dementia, i.e., those eligible for anti-amyloid therapy [10, 15].

We hypothesized that patients with mixed AD and non-AD co-pathologies, the latter referred to as AD^+^ from hereon, would exhibit more substantial hippocampal atrophy, while ‘pure’ AD would be associated with greater atrophy in AD-typical cortical regions. Furthermore, we postulated that patients with AD^+^-classified atrophy would show more significant memory impairment and accelerated clinical decline. At the same time, those with a ‘pure’ AD-classified GM profile would progress more slowly but exhibit more non-memory cognitive deficits, e.g., in the executive and language domains. Additionally, we sought to determine the degree to which cortical GM measures, reflecting tau pathology, and hippocampal GM, reflecting non-AD co-pathologies, are associated with future clinical progression.

## Methods

Data used in the preparation of this article were obtained from the Alzheimer’s Disease Neuroimaging Initiative (ADNI) database (adni.loni.usc.edu). The ADNI was launched in 2003 as a public–private partnership, led by Principal Investigator Michael W. Weiner, MD. The primary goal of ADNI has been to test whether serial magnetic resonance imaging (MRI), positron emission tomography (PET), other biological markers, and clinical and neuropsychological assessment can be combined to measure the progression of mild cognitive impairment (MCI) and early Alzheimer’s disease (AD). For up-to-date information, see www.adni-info.org.

### Participants

#### Derivation cohort

We used the subsample of ADNI participants who had undergone post-mortem neuropathological examinations to identify atrophy patterns indicative of ‘pure’ AD or mixed pathology. This ‘derivation cohort’ (downloaded on February 9th, 2024) comprised 110 participants ranging from cognitively normal to dementia. Thereof, all participants who had undergone an MRI measurement at the stage of MCI or mild dementia, i.e., those with an MMSE of 20 or greater [16], and who had complete post-mortem data regarding AD-, limbic TDP-43, and AGD-pathology, were selected for the analysis (*n =* 91). For each patient, we selected the last MRI at this level of impairment to reduce the latency between the MRI measurement and the histopathological assessment. These participants were then classified according to their neuropathological profiles as described below (please see *Neuropathological classification*).

#### Validation cohort

To validate the utility of the MRI measures derived from the derivation cohort, ADNI patients at the stage of MCI (*n =* 224) and mild dementia (*n =* 221) with CSF markers indicative of AD pathology [17], who were not part of the derivation cohort, were selected as the validation cohort.

#### Neuropathological classification

Standardized neuropathological assessments of the autopsied ADNI participants are provided by the ADNI Neuropathology Core [18]. Following the National Institute on Aging-Alzheimer’s Association guidelines, AD neuropathologic change (ADNC) was categorized as absent, low, intermediate, or high, depending on the Thal amyloid beta (Aβ) phases, Braak neurofibrillary tau tangle stages, and the Consortium to Establish a Registry for Alzheimer’s Disease (CERAD) score, rating the density of neuritic plaques [19]. TDP-43 pathology is described based on the presence or absence of TDP-43-immunoreactive inclusions in the spinal cord, the amygdala, the hippocampus, the entorhinal cortex/inferior temporal gyrus, and the frontal neocortex [20]. Limbic TDP-43 pathology was defined as TDP-43 inclusions in the amygdala, the hippocampus, or the entorhinal cortex/inferior temporal gyrus region [2]. Additionally, the presence of AGD and other tauopathies was reported [21].

### Imaging data

#### Magnetic resonance imaging acquisition

ADNI MRI data were acquired on 1.5 and 3 T MRI scanners by Siemens, Philips, and General Electric Healthcare. For our analysis, scanner-specific 3D sagittal T_1_-weighted magnetization-prepared rapid gradient-echo (MPRAGE) sequences were employed. ADNI’s original MPRAGE sequences undergo standardized image correction steps during preprocessing to increase signal uniformity across different scanner types and trial centers. The ADNI webpage (https://adni.loni.usc.edu) provides a detailed description of the ADNI imaging data acquisition and standardized preprocessing steps.

#### Magnetic resonance imaging processing

We assessed GM volumes using voxel-based morphometry (VBM) implemented in the SPM toolbox CAT12 [22] (https://www.neuro.uni-jena.de/cat). Specifically, T_1_-images were denoised, using a spatially adaptive nonlocal means filter, resampled to an isotropic voxel size, and underwent bias field correction. The resulting images were then segmented into tissue classes and spatially normalized into Montreal Neurological Institute (MNI) space using the high-dimensional registration algorithm DARTEL [23]. The resulting normalized GM maps were modulated only for nonlinear normalization to account for global differences in head size modeled by the linear transform. Average voxel intensities were extracted from the modulated normalized GM maps using masks in MNI space corresponding to the regions of interest (ROIs) described below.

#### Derivation of pathology-specific gray matter changes

**Fig. 1:**
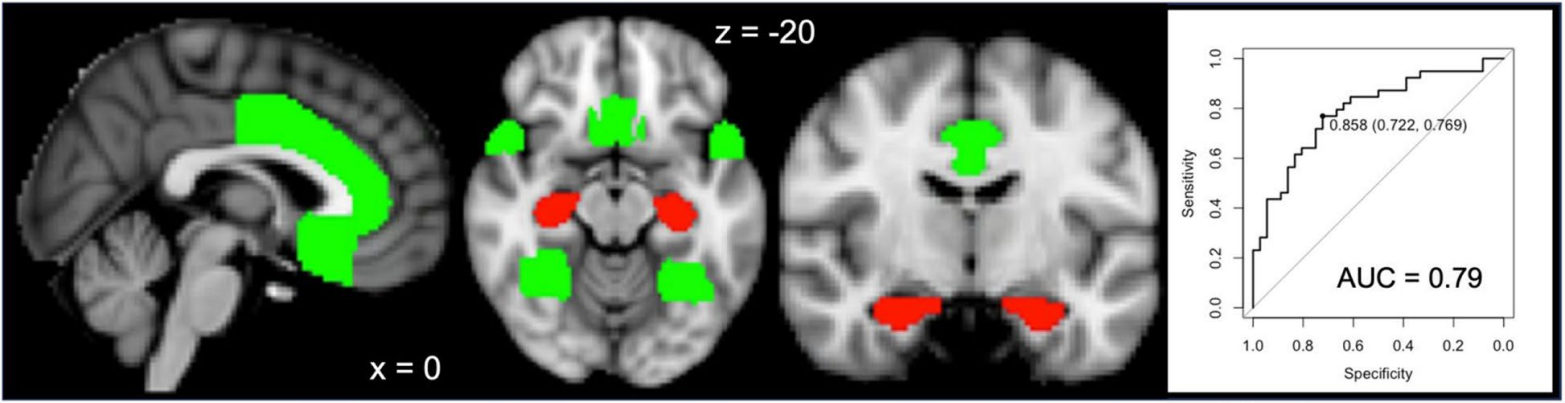
Cortex-to-hippocampus ratio discriminates non-AD co-pathologies. Gray matter of the anterior cingulate, the subcallosal gyrus, the temporal pole, and the fusiform gyrus (green) divided by the volume of the hippocampus (red) was significantly smaller (*p* < 0.005, Bonferroni-corrected at the ROI level) in patients with ‘pure’ AD pathology, compared to patients with AD and limbic non-AD co-pathology, i.e., AD^+^. The ratio of GM in the aforementioned cortical areas relative to hippocampal GM discriminated between AD and AD.^+^ with an area under the curve (AUC) of 0.79 at a cut-off of 0.858. At this cut-off, the sensitivity for non-AD co-pathologies was 0.769, with a specificity of 0.722

To generate a GM measure that discriminates between ‘pure’ AD and AD^+^ patients at relatively early disease stages, ROIs were identified where GM differed significantly between the two groups. To minimize bias towards the typical AD regions in the lateral temporo-parietal cortices and avoid overlooking areas associated with earlier Braak stages, we examined all cortical regions defined in the Harvard–Oxford anatomical atlas [24] and the hippocampus. The atlas ROIs were thresholded at 25% probability. To improve the discrimination between the groups, analogous to previous studies investigating AD subtypes and AD-typicality [2, 13, 25–27], for each cortical ROI, the regional GM was divided by the hippocampal GM. Since the resulting GM ratios of several ROI were not normally distributed, 2-sample Wilcoxon tests were performed to compare the ROI ratios between the groups. After Bonferroni correction for multiple comparisons (p < 0.005), significant differences between ‘pure’ AD and AD^+^ patients were observed for the ratio between regional GM and hippocampal GM for the fusiform gyrus, the anterior cingulate cortex, the subcallosal cortex, and the temporal pole (Fig. 1). The average GM volume of these four cortical regions relative to hippocampal GM was then examined regarding its utility in discriminating between the groups (see *Statistical Analysis* below).

To capture the AD-typical cortical tau-protein burden in an MRI measure, GM volume was averaged across a cortical AD meta region consisting of the angular gyrus, the posterior cingulate cortex, and the inferior temporal gyrus [26, 28]. In the derivation cohort, a Spearman correlation was computed between the GM volume of the AD meta region and the post-mortem Braak stage to verify the association between GM volume in this region and tau pathology.

#### Neuropsychological assessment

We used performance on the Mini-Mental Status Exam (MMSE) to measure global cognition. The clinical dementia rating – sum of boxes (CDR-SB) was included to reflect the severity of clinical disease symptoms. Of the neuropsychological assessments available from the ADNI patients, episodic memory delayed recall (delayed recall of the logical memory (LM-DR) and the percentage of words forgotten after delay on the Rey auditory verbal learning test (RAVLT % forgotten) were used to reflect hippocampal function. The cortical function was captured via assessments of language (verbal fluency for the category animals (CATANIM) and the Boston naming test (BNT)), visuospatial function (the clock-drawing test) and executive function (the trail-making tests (TMT) A and B).

The annual rate of changes in the MMSE and the CDR-SB between baseline and the last follow-up were computed for each participant to quantify clinical progression.

### Statistical analysis

To identify the cut-off for the cortex-to-hippocampus ratio that optimally discriminates between ‘pure’ AD and AD^+^ in the derivation cohort, a receiver operating characteristic (ROC) analysis was performed as implemented in the ‘R’-package ‘pROC’. Based on this cut-off for the cortex-to-hippocampus ratio, patients of the in vivo validation cohort were classified into two groups: a ‘pure’ AD-classified group with a ratio below, and an AD^+^-classified group with a ratio above the cut-off.

We then examined in each cohort whether groups differed in neuropsychological performance or age. This was done using the Wilcoxon rank-sum test, as several of these measures were not normally distributed.

Furthermore, we performed a multiple regression analysis to examine the association of degeneration of specific cerebral structures with measures of clinical progression in the in vivo validation cohort. Specifically, the annual rates of change in MMSE and CDR-SB during the follow-up were defined as dependent variables. GM of the cortical AD meta-ROI and the hippocampus, as well as age, gender, and education, were entered as independent variables. This analysis was performed separately in the MCI and the mild dementia groups of the validation cohort to probe disease-stage-specific associations.

## Results

### Demographics and disease stage

In the derivation cohort, AD patients did not differ from AD^+^ patients in age, gender distribution, or latency between MRI and death (Table 1). The average latency between the MRI and death was 4.49 ± 2.77 years. A total of 75 patients had AD, as evidenced by intermediate to high ADNC. Of these, 39 (52%) were AD^+^, i.e., had comorbid limbic TDP-43 pathology and/or AGD, while 36 (48%) had relatively ‘pure’ AD pathology. The majority of patients in both groups were at the stage of mild dementia (AD 34/36, AD^+^ 37/39) at the time of MRI, with two patients in each group being at the stage of MCI.

**Table 1:** Demographics and neuropsychological characteristics of the derivation cohort

Derivation cohort						
	AD (*n =* 36)		AD & TDP-43/AGD (*n =* 39)		Group difference	
MCI/dementia	2/34		2/37			
Gender (m/f)	26/10		29/10			
	mean	sd	mean	sd	W	*p*-value
Age at MRI	77.27	7.54	79.99	7.39	551.5	0.112
Latency MRI to death	4.08	2.67	4.87	2.85	577.5	0.185
MMSE	23.94	2.35	24.1	2.62	695	0.944
CDR-SB	4.7	2.15	4.38	2.22	719.5	0.549
Logical Memory DR	3.55	4.19	1.97	3.32	624	0.059
Verbal memory forgetting (%)	86.67	24.05	95.65	11.88	533	**0.031**
TMT-A	65.44	38.14	52	25.77	802.5	0.289
TMT-B	204.69	91.99	193	97.37	711	0.617
Boston Naming Test	23.56	5.96	22.32	5.88	728	0.173
Verbal fluency (animals)	12.92	5.42	11.79	5.36	770	0.473
Clock-drawing test	3.64	1.38	3.62	1.23	726.5	0.793

*AD* Alzheimer’s disease, *AGD* Argyrophilic grain disease, *sd* Standard deviation, *CDR-SB* Clinical dementia rating—sum of the boxes, *DR* Delayed recall, *MCI* Mild cognitive impairment, *MMSE* Mini-Mental Status Exam, *TMT* Trail-Making-Test

Of the validation cohort, 144 of the 224 MCI patients (64%) had a cortex-to-hippocampus ratio < 0.858, suggestive of a ‘pure’ AD pathology. In contrast, 80 (36%) had a greater cortex-to-hippocampus ratio, suggestive of mixed, AD^+^ pathology. Of the 221 patients with mild dementia in the validation cohort, 104 (47%) had a cortex-to-hippocampus ratio suggestive of ‘pure’ AD pathology. In contrast, the ratio was suggestive of AD^+^ in the remaining 117 (53%) patients. At both disease stages, AD^+^-classified patients were older than ‘pure’ AD-classified patients (Table 2).

**Table 2:** Demographics and neuropsychological characteristics of the validation cohort

Mild Dementia (MMSE > 20)								
	AD-classified			AD^+^-classified			Group Difference	
	n	mean	sd	n	mean	sd	W	*p*-value
Gender (male/female)	54/50			72/45				
Age	104	**73.52**	8.46	117	**76.26**	6.77	4958	**0.018**
ΔMMSE per year	97	−2.70	2.96	114	−2.37	2.77	5247.5	0.525
ΔCDR-SB per year	97	1.70	1.67	111	1.61	1.72	5714	0.446
MMSE	104	23.63	2.02	117	23.21	1.69	6717.5	0.176
CDR-SB	104	4.90	1.76	117	4.77	1.73	6435	0.457
Logical Memory DR	92	1.85	2.74	97	1.08	2.00	5180	**0.033**
Verbal memory forgetting (%)	104	86.74	24.90	114	94.37	15.22	5070.5	**0.012**
TMT-A	104	69.64	37.56	115	57.03	31.37	7126	**0.014**
TMT-B	97	212.03	94.88	112	194.83	85.38	6086	0.127
Boston naming test	104	22.19	6.83	114	22.01	5.46	6442	0.269
Verbal fluency (animals)	104	11.43	4.58	116	12.53	4.69	5150.5	0.061
Clock-drawing test	104	3.28	1.35	116	3.43	1.45	5561	0.306
**Mild cognitive impairment**								
	AD-classified			AD^+^-classified			Group Difference	
	n	mean	sd	n	mean	sd	W	*p*-value
Gender (male/female)	84/60			52/28				
Age	144	**73.68**	7.35	80	**77.24**	6.28	4071.5	**< 0.001**
ΔMMSE per year	131	**−1.04**	1.74	80	**−1.73**	2.12	6230	**0.021**
ΔCDR-SB per year	126	**0.69**	0.98	79	**1.01**	1.15	3826	**0.005**
MMSE	144	26.93	2.13	80	26.56	1.88	6416	0.154
CDR-SB	142	1.53	0.89	80	1.81	1.06	4849.5	0.067
Logical Memory DR	129	**5.47**	3.95	75	**3.79**	3.77	6098	**0.002**
Verbal memory forgetting (%)	144	**64.13**	52.66	80	**78.78**	25.43	4563	**0.008**
TMT-A	144	45.31	22.88	80	47.46	23.29	5481	0.549
TMT-B	143	128.64	73.79	78	138.27	67.57	4798.5	0.087
Boston naming test	143	25.62	4.32	77	25.1	3.84	6220	0.129
Verbal fluency (animals)	144	16.25	5.15	80	16.09	4.82	5730	0.949
Clock-drawing test	144	4.28	0.92	80	4.2	1.04	5899.5	0.743

*AD* Alzheimer’s disease, *AGD* Argyrophilic grain disease, *sd* standard deviation, *CDR-SB* Clinical dementia rating—sum of the boxes, *DR* Delayed recall, *MCI* Mild cognitive impairment, *MMSE* Mini-Mental Status Exam, *TMT* Trail-Making-Test

### Neuropsychological findings in the derivation cohort

In the derivation cohort, verbal episodic memory (percentage forgotten on the RAVLT) was poorer in patients with mixed pathology (W = 533, *p =* 0.031), and there was a trend towards poorer logical memory, reflected in a lower logical memory delayed recall (W = 624, *p*-value = 0.059). The groups did not differ with respect to verbal fluency, naming, visuospatial function, or performance on the trail making tests (all *p*-values > 0.17, cf. Table 1).

### Co-pathologies are associated with greater hippocampal and less cortical atrophy in the derivation cohort

Less hippocampal GM was observed in patients with mixed pathology than those with ‘pure’ AD pathology (AD^+^ 0.57 ± 0.09, AD 0.63 ± 0.1, *p =* 0.011). Conversely, GM in the fusiform gyrus (AD^+^ 0.61 ± 0.04, AD 0.58 ± 0.08, *p =* 0.02) and the middle frontal gyrus (AD^+^ 0.42 ± 0.05, AD 0.4 ± 0.04, *p =* 0.044) was less in patients with ‘pure’ AD than in those with mixed pathology.

### The cortex-to-hippocampus ratio differs significantly between AD and AD^+^ in the derivation cohort

In patients with mixed pathology compared to patients with ‘pure’ AD pathology, after Bonferroni-correction for multiple comparisons, significantly greater ratios of cortical GM relative to hippocampal GM were observed for the fusiform gyrus (AD^+^ 1.11 ± 0.19, AD 0.93 ± 0.13, *p =* 0.001), the anterior cingulate cortex (AD^+^ 0.92 ± 0.14, AD 0.81 ± 0.09, *p =* 0.004), the subcallosal cortex (AD^+^ 1.05 ± 0.15, AD 0.92 ± 0.13, *p =* 0.004), and the temporal pole (AD^+^ 0.87 ± 0.1, AD 0.76 ± 0.12, *p =* 0.004). ROC analyses indicated that the cortex-to-hippocampus ratio of GM, averaged over these four regions, discriminated between AD and AD^+^ patients with an AUC of 0.79 when thresholded at 0.858 (Fig. 1).

### Alzheimer-typical temporo-parietal atrophy correlates with the post-mortem Braak stage in the derivation cohort

**Fig. 2:**
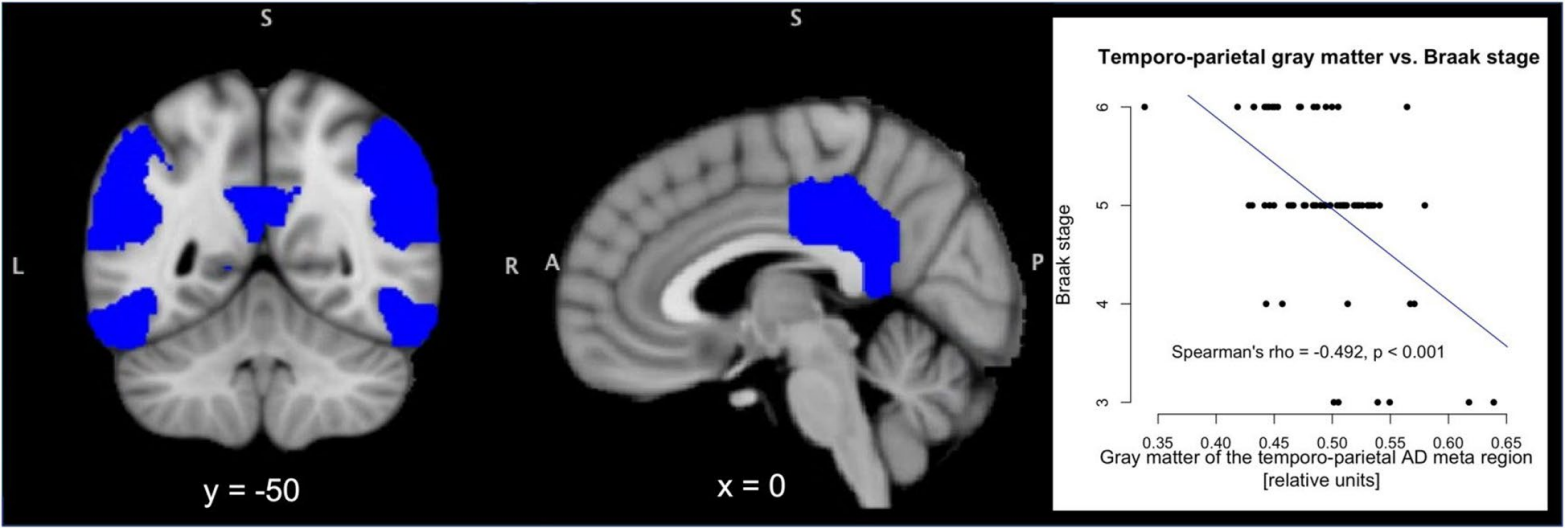
Gray matter of the temporo-parietal cortex is negatively correlated with post-mortem Braak stages. The gray matter of the temporo-parietal Alzheimer’s disease (AD) meta region of interest (blue) showed a negative correlation with the post-mortem Braak stages across the AD patients of the derivation cohort (*n =* 75, Spearman’s rho = −0.492, *p* < 0.001). y and z denote slice coordinates in Montreal Neurological Institute space

GM volume of the AD meta-ROI consisting of the inferior temporal gyrus, the posterior cingulate cortex, and the angular gyrus exhibited a significant negative correlation with the post-mortem Braak stage across the patients in the post-mortem derivation cohort (Spearman’s rho = −0.492, *p* < 0.001; Fig. 2).

### Cognitive profiles of ‘pure’ AD-classified and AD^+^*-*classified patients in the validation cohort

AD^+^-classified patients exhibited significantly poorer episodic memory function than ‘pure’ AD-classified patients, especially at the stage of MCI, but also in mild dementia (Table 2). Furthermore, at the stage of mild dementia, ‘pure’ AD-classified patients were significantly slower on the TMT-A. There was also a non-significant trend towards poorer verbal fluency in ‘pure’ AD-classified patients at the stage of mild dementia. No significant differences in language, visuospatial, or executive function were observed between ‘pure’ AD-classified and AD^+^-classified patients.

### More rapid clinical progression in AD^+^*-*classified patients at the stage of MCI

92% of MCI (*n =* 207/224) patients had at least one follow-up assessment with an average time to the last follow-up of 3.79 ± 2.87 years, while follow-up data were available from 94% of the patients with mild dementia (*n =* 207/221), with an average time to the last follow-up of 1.95 ± 1.39 years. Patients with MCI and AD^+^-classified atrophy exhibited more rapid clinical progression during the follow-up period, indicated by a more significant annual decrease in the MMSE and a more substantial annual increase in the CDR-SB, compared to patients with a “pure”-AD like atrophy (MMSE: AD^+^-classified −1.73 ± 2.12 points per year, AD-classified −1.04 ± 1.74 points per year, *p =* 0.021; CDSB: AD^+^-classified 1.01 ± 1.15 points per year, AD-classified 0.69 ± 0.98 points per year, *p =* 0.005; Table 2**,** bottom). However, at the stage of mild dementia, no difference between AD^+^-classified and ‘pure’ AD-classified patients was observed regarding clinical progression during follow-up.

### Predictors of clinical progression in MCI and mild dementia

Multiple regression analysis, adjusted for age, gender, and education, revealed that GM of the AD meta-ROI, rather than hippocampal GM, predicted the annual rate of change on the MMSE and the CDR-SB during the follow-up, both in MCI and mild dementia. It was only at the stage of MCI that higher age was also weakly associated with a slower annual decline of the MMSE during follow-up (Table 3).

**Table 3:** MRI based predictors of clinical progression in MCI and mild dementia

Mild cognitive impairment						95% CI		Model statistics	
**Dependent variable**	**Explanatory variables**	**Unstandardized betas**	**Standard Error**	**t**	***p***	**Lower**	**Upper**	**R**	***R***^**2**^
Change in **MMSE** per year*	**AD meta GM**	**15.078**	**3.322**	**4.54**	**<.001**	**8.529**	**21.627**	0.405	0.164
	Hippocampal GM	2.573	1.775	1.449	0.149	−0.927	6.073		
	**Age**	**0.041**	**0.019**	**2.144**	**0.033**	**0.003**	**0.079**		
	Education	−0.015	0.044	−0.349	0.727	−0.103	0.072		
	Gender	−0.035	0.264	−0.132	0.895	−0.556	0.486		
Change in **CDR-SB** per year*	**AD meta GM**	**−6.56**	**1.917**	**−3.423**	**<.001**	**−10.34**	**−2.78**	0.343	0.118
	Hippocampal GM	−1.701	1.008	−1.687	0.093	−3.689	0.287		
	Age	−0.015	0.011	−1.359	0.176	−0.036	0.007		
	Education	−0.002	0.025	−0.069	0.945	−0.052	0.048		
	Gender	−0.045	0.152	−0.297	0.767	−0.344	0.254		
**Mild dementia**						**95% CI**		**Model statistics**	
**Dependent variable**	**Explanatory variables**	**Unstandardized betas**	**Standard Error**	**t**	***p***	**Lower**	**Upper**	**R**	***R***^**2**^
Change in **MMSE** per year*	**AD meta GM**	**20.078**	**4.303**	**4.667**	**<.001**	**11.595**	**28.561**	0.351	0.123
	Hippocampal GM	0.018	0.028	0.642	0.522	−0.037	0.073		
	Age	−0.083	0.068	−1.22	0.224	−0.218	0.051		
	Education	−1.147	2.339	−0.49	0.624	−5.76	3.465		
	Gender	0.363	0.4	0.908	0.365	−0.425	1.151		
Change in **CDR-SB** per year*	**AD meta GM**	**−9.157**	**2.621**	**−3.493**	**<.001**	**−14.326**	**−3.989**	0.275	0.076
	Hippocampal GM	0.022	0.017	1.289	0.199	−0.012	0.056		
	Age	0.043	0.042	1.037	0.301	−0.039	0.125		
	Education	0.42	1.434	0.293	0.77	−2.408	3.248		
	Gender	−0.249	0.244	−1.019	0.309	−0.73	0.233		

*AD* Alzheimer’s disease, *GM* Gray matter, *CI* Confidence interval, *MMSE* Mini mental status exam, *CDR-SB* Clinical dementia rating sum of boxes

^*^Mean follow-up duration was 3.79 ± 2.87 years in MCI and 1.95 ± 1.39 years in mild dementia

## Discussion

The present data indicate that patients with ‘pure’ AD pathology differ from those with AD^+^, i.e., AD neuropathological changes and limbic non-AD co-pathology, concerning hippocampal volume and cortical GM atrophy. Notably, the ratio of GM of cortical areas associated with intermediate Braak stages, relative to hippocampal GM, differed significantly between these two types of AD patients. Furthermore, the application of this ratio to an independent sample demonstrated that an AD^+^-classified atrophy pattern was associated with more substantial memory impairment and, at the stage of MCI, a more rapid clinical progression in the following years. However, this was not the case at the stage of mild dementia, where ‘pure’ AD-classified and AD^+^-classified patients no longer differed regarding clinical progression.

‘Pure’ AD patients in the derivation sample exhibited less hippocampal atrophy and greater cortical GM atrophy relative to hippocampal GM, predominantly in the fusiform gyrus, but also in the anterior cingulate cortex and the adjacent subcallosal cortex, as well as the temporal pole. In the context of AD progression, these areas are part of the intermediate Braak stages III (fusiform gyrus) and IV (anterior cingulate cortex, subcallosal cortex, and temporal pole) [29, 30]. Tau accumulation in these areas is observed at the clinical stages of MCI and mild dementia [31]. Hence, under the hypothesis that the effects of AD and non-AD co-pathologies, reflected in cortical and hippocampal degeneration, on cognition are cumulative [9], it is conceivable that ‘pure’ AD, which entails less hippocampal atrophy than AD^+^, would result in more extended tau pathology and atrophy in cortical intermediate Braak stage areas in MCI and mild dementia. In other words, cortical degeneration appears to be less severe in the presence of the predominantly temporomesial non-AD co-pathologies, because the AD-pathology does not need to reach the severity seen in ‘pure’-AD to result in a similar overall level of impairment.

The cortex-to-hippocampus ratio generated by averaging regional GM over the four cortical regions and dividing by hippocampal GM discriminated between AD and AD^+^ patients in the derivation cohort with an AUC of 0.79. This finding is in the range of previously reported values discriminating between ‘pure’ AD and ‘pure’ LATE patients [2, 14, 32], albeit being substantially higher than previously reported values for the discrimination between AD and AD^+^ pathologies with an inferior to medial temporal ratio of GM [14]. This increase in discriminatory capability may be attributable to the fact that the present ROI were derived from the direct comparison of ‘pure’ AD and AD^+^ patients, highlighting the fusiform gyrus, which is affected by AD before the inferior and middle temporal gyri, commonly used in these ratios. Further, with the clinical application in mind, we specifically constrained the analysis to MCI and mild dementia. In contrast, previous investigations included severely impaired patients [2, 32], which may have lowered the sensitivity to more subtle atrophy patterns.

Applying our cortex-to-hippocampus ratio in the in vivo validation sample yielded findings in line with previous reports of more rapid clinical progression and more severe cognitive impairment in AD^+^ as compared to ‘pure’ AD pathology [3, 6, 7]. The number of ‘pure’ AD-classified patients at the stage of MCI was almost twice that of AD^+^-classified patients, while the distribution was relatively even in mild dementia, indicating more severe impairment in AD^+^ as compared to ‘pure’ AD. Furthermore, AD^+^-classified patients at the stage of MCI exhibited significantly faster clinical progression than those with a ‘pure’ AD-classified atrophy. We did not observe this effect in mild dementia. A possible explanation may lie in the shorter average follow-up in dementia compared to MCI. It is also conceivable that the hippocampal degeneration, which contributes substantially to the cortex-to-hippocampus ratio and which was used to define the two groups, was already near its maximum at the stage of mild dementia in both AD and AD^+^ [31], thus contributing less to the future cognitive decline. This explanation was corroborated by the observation that the difference in memory performance between ‘pure’ AD-classified and AD^+^-classified patients was less pronounced in mild dementia than MCI.

In general, aside from the fact that an AD^+^-classified atrophy pattern was associated with faster clinical progression, the present data linking GM in the temporo-parietal AD meta-ROI to post-mortem Braak stage, deterioration of global cognition, and increasing clinical severity, suggest that the primary driver of clinical progression is cortical degeneration. This suggestion is supported by PET studies demonstrating the association between dementia and later Braak stages [31] and linking cortical tau pathology to subsequent cognitive decline [33]. Cortical tau pathology, in turn, has been linked to cortical atrophy, especially in lateral temporal areas [34, 35], which are also part of the AD meta-ROI employed in the current work.

### Limitations

The cortex-to-hippocampus ratio presented here to differentiate between ‘pure’ AD-classified and AD^+^-classified AD patients was derived from post-mortem data and applied to an independent sample. While the distribution of ‘pure’ AD-classified and AD^+^-classified in the independent in vivo sample is plausible, and the faster clinical progression in AD^+^-classified patients aligns with previous findings from post-mortem cohorts [6, 7], a reproduction of the classification accuracy of the cortex-to-hippocampus ratio in a separate post-mortem sample would strengthen the case for the pathological correlates of this measure.

Another limitation is that most patients in the present ADNI dataset had a typical amnestic AD phenotype. It therefore remains unclear whether our findings regarding the association between a greater cortex-to-hippocampus ratio and more rapid disease progression are also applicable to atypical AD variants, which often spare the medial temporal lobe, but still present with a rapid clinical progression.

Furthermore, only nuanced differences between ‘pure’ AD and AD^+^ patients regarding cortical function were detectable. This finding is, at least in part, attributable to the retrospective nature of this investigation, which limited the analyses to available neuropsychological data. Future investigations are warranted to explore whether ‘pure’ AD patients at the stage of MCI or mild dementia may already exhibit more substantial impairment than AD^+^-patients at the same stage in cognitive functions specific to the fusiform gyrus and other inferior temporal areas, e.g., facial and object recognition [36, 37].

## Conclusions

The current data suggest that the cortex-to-hippocampus ratio encompassing intermediate Braak stage cortical regions may be a powerful tool for detecting non-AD co-pathologies in vivo and facilitating a more accurate prognosis in AD at the MCI stage. Furthermore, the data underscore that the prognostic value of medial temporal lobe atrophy in MCI, and especially in mild dementia due to AD, is limited. Instead, cortical atrophy closely related to tau pathology may better indicate clinical progression. These imaging markers could also prove helpful in predicting the response to anti-amyloid therapies, as they are more readily available than tau PET.

## Data Availability

The datasets used and/or analyzed during the current study are available from the ADNI database.

## Abbreviations

ADNI: Alzheimer’s disease neuroimaging initiative
ADNC: Alzheimer’s disease neuropathologic change
AGD: Argyrophilic grain disease
BNT: Boston naming test
CATANIM: Verbal fluency
CDR-SB: Clinical dementia rating Sum of boxes
CSF: Cerebrospinal fluid
CERAD: Consortium to Establish a Registry for Alzheimer’s Disease
GM: Gray matter
LANS: Limbic amnestic neurodegenerative syndromes
LATE: Limbic predominant age-related TDP-43 encephalopathy
LM-DR: Logical memory: delayed recall
MCI: Mild cognitive impairment
MMSE: Mini-Mental Status Exam
MPRAGE: Magnetization-prepared rapid gradient-echo
MRI: Magnetic resonance imaging
MNI: Montreal Neurological Institute
PET: Positron emission tomography
RAVLT: Rey auditory verbal learning test
ROC: Receiver operating characteristic
ROI: Region of interest
TMT: Trail-making-test
VBM: Voxel-based morphometry
